# The relationship between clinical work stress and anxiety in master’s degree nursing students: The mediating role of psychological capital and social support

**DOI:** 10.1097/MD.0000000000033997

**Published:** 2023-06-09

**Authors:** Chunguang Ling, Shaojie Yu

**Affiliations:** a Faculty of Education, Qufu Normal University, Qufu, P.R. China; b School of Graduate, Weifang Medical University, Weifang, P.R. China; c School of Psychology, Weifang Medical University, Weifang, P.R. China.

**Keywords:** anxiety, clinical work pressure, mediation, postgraduate students of nursing

## Abstract

Work stress and anxiety are major problems faced by graduate nursing students. Research on the relationships between these factors has the potential to improve the psychological state of graduate nursing students. This study gathered a valid sample of 321 graduate nursing students and performed structural equation modeling and multiple regression to test the proposed research model. The study used the Clinician Work Stress Scale, Psychological Capital Scale, Social Support Rating Scale, and State-Trait Anxiety Scale to survey the sample. Correlation analysis showed that job stress was significantly correlated with psychological capital (*r *= −0.46, *P* < .01), social support (*r *= −0.21, *P* < .01), and anxiety (*r *= 0.47, *P* < .01). Psychological capital (*r *= −0.56, *P* < .01) and social support (*r *= −0.43, *P* < .01) were significantly correlated with anxiety. The results of the path analysis showed that psychological capital (0.21, 95% confidence interval: 0.19–0.39) and social support (0.07, 95% CI: 0.02–0.15) played a mediating role in the relationship between job stress and anxiety, and the mediating effect accounted for a percentage of the total effect (51.85%). There is a direct relationship between clinical social work stress and anxiety among nursing postgraduates. Anxiety is significantly reduced through the intermediary effects of psychological capital and social support.

## 1. Introduction

The Academic Degrees Committee of the State Council’s “Guiding Training Program for Master of Nursing Students” states that the Master of Nursing students should grasp the ability to perform standardized clinical nursing operations and deal with nursing problems independently in their specialty. Master of Nursing students conduct clinical practice at the front line of the clinic; at the same time, they are also required to complete many tasks such as scientific research and graduation dissertations. To some extent, multi-dimensional work pressures can increase the level of an individual’s anxiety. It has been shown that excessive work stress can have adverse effects on the physiology, psychology, behavior, and cognition of clinical personnel.^[1]^ Research shows that anxiety is common among nurses in China, with a rate of 75.2%.^[2]^ Therefore, it is important to study the mechanisms of anxiety formation in master’s degree nursing students and explore the factors influencing anxiety formation.

In the field of psychological research, the meaning of work stress has been interpreted in different ways: some focus on the origin of pressure, which is the reflection of work beyond normal bounds; while others focus on the operability of the definition of pressure. This perspective asserts that pressure includes the workload, work complexity, role conflict, role ambiguity, and so on.^[3]^ Based on the above studies, this research defines work stress as workers engendering psychological changes and behavioral reactions under the influence of stressors. With regard to persistent work stress, anxiety may occur when there is an imbalance between personal effort and feedback.^[4]^ Based on the above analysis, this study proposed hypothesis H_1_: Clinical work stress among master’s degree nursing students can directly affect anxiety.

Psychological capital originates from the research field of positive psychology, and refers to the positive psychological state of an individual in the process of growth and development. It encompasses 4 dimensions: self-efficacy, optimism, resilience, and hope.^[5]^ In this study, psychological capital is the positive psychological state of individuals and a psychological resource to promote personal growth. In reality, different individuals facing the same work stress show different levels of anxiety. Some studies have shown that psychological capital plays a mediating role between various work stressors and emotional responses; some studies have also suggested that they can alleviate an individual’s anxiety by developing the psychological capital of individuality and organization.^[6]^ Based on the above analysis, this study proposes hypothesis H_2_: Psychological capital of master’s degree nursing students plays a mediating role between job stress and anxiety.

**Figure 1.**
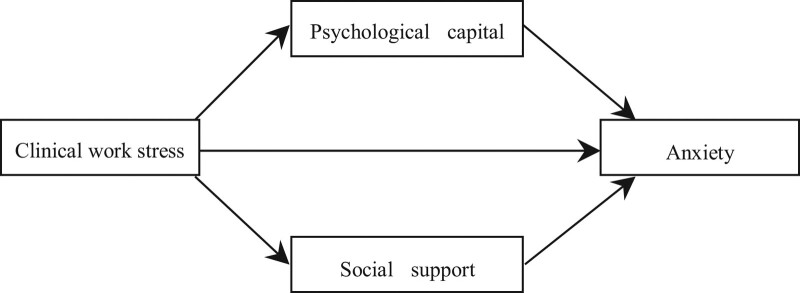
Assumption model.

Social support refers to the resources obtained by people’s social connections, which can reduce psychological stress, relieve mental tension, and improve social adaptability.^[7]^ Social support can reduce the working pressure on medical staff to some extent, and improve their mental health, and has played a very important role in improving the quality of life of medical staff.^[8]^ Social support can improve an individual’s level of hope, enhance their feeling of being respected, and relieve individual anxiety.^[9]^ The increase in social interaction skills in the social support system can effectively reduce the level of individual social anxiety.^[10]^ Based on the above analysis, this study proposed hypothesis H_3_: Social support for master’s degree nursing students plays a mediating role between job stress and anxiety. Figure 1 shows the assumption model.

Existing studies have also analyzed the relationship between clinical work stress and anxiety but have not considered psychological capital and social support as intermediaries. This study integrates psychological capital and social support into the work stress-anxiety model (shown in Fig. 1) and constructs a new way to reduce anxiety through psychological capital and social support in clinical work stress situations.

## 2. Objectives and methods

### 2.1. Sample

Master’s degree nursing students from 5 medical universities (Weifang Medical University, Xuzhou Medical University, Gannan Medical University, Chongqing Medical University, and Wenzhou Medical University) in China were selected as research participants simultaneously from September 15 to September 20, 2022. We selected 1 class each from grades one to three, and only students with odd student ID were allowed to answer the questionnaire. The inclusion criteria were clinical work experience of more than 3 months, and willingness to complete the questionnaire. In total, 343 questionnaires were distributed online using a Questionnaire Star. A total of 343 questionnaires were returned, and invalid questionnaires (missed and regular answers) were manually excluded, resulting in 321 valid questionnaires with an effective response rate of 93.59%. Among the valid respondents, 23.91% were male and 76.09% were female. The sample size calculation formula (N = 400 Q/P, N = 400 (1–77.3%)/77.3%, and N = 118) showed that the sample size of this study was sufficient. Table 1 presents the participants’ demographic variables.

**Table 1:** Demographic variables of samples.

Variable	Dimension	Number	Percent
Gender	Male	77	23.91
	Female	244	76.09
Age	20–25	264	82.24
	26–30	40	12.46
	Older than 30	17	5.30
Grade	1	90	28.04
	2	105	32.71
	3	126	39.25
Family origin	Urban	128	39.87
	Rural	193	60.13

### 2.2. Methods

#### 2.2.1. The Scale for Occupational Stressors on Clinicians.

The Scale for Occupational Stressors on Clinicians was developed by Chen J. in 2009 and consists of 7 dimensions: organizational management, career interest, workload, career development, interpersonal relationship, external environment, and doctor-patient relationship, with 38 questions in total and scored 1 to 4 on a Likert 4-point scale.^[11]^ In this study, the Cronbach’s α coefficient was 0.923. The Kaiser-Meyer-Olkin value was 0.826, and Bartlett’s test of sphericity produced an *x* value of 9411.7 (*P* < .01).

#### 2.2.2. The Positive Psychological Capital Questionnaire.

The Positive Psychological Capital Questionnaire was developed by Zhang K. in 2010 and consists of 4 dimensions: self-efficacy, resilience, hope, and optimism, with a total of 26 questions and scored 1 to 5 on a Likert 5-point scale.^[12]^ In this study, the Cronbach’s α coefficient for this scale was 0.929. The Kaiser-Meyer-Olkin value was 0.891, and Bartlett’s test of sphericity produced an *x*-value of 8411.7 (*P* < .01).

#### 2.2.3. The Social Support Rating Scale.

The Social Support Rating Scale was developed by Xiao SH in 1994, and consists of 3 dimensions: subjective support, objective support, and support utilization, with a total of 10 questions scored from 1 to 4 on a 4-point scale. The higher the score, the higher the social support.^[13]^ In this study, the Cronbach’s α coefficient for this scale was 0.920. The Kaiser-Meyer-Olkin test value was 0.824, and Bartlett’s test of sphericity produced an *x*-value of 9121.7 (*P* < .01).

#### 2.2.4. The State-Trait Anxiety Inventory.

The State-Trait Anxiety Inventory was compiled by Charles Spielberger in 1977 and revised in 1983 to 40 items; the first 20 items were the State Anxiety Inventory and the last 20 items were the Trait Anxiety Inventory.^[14]^ This study only used the Trait Anxiety Inventory which was scored from 1 to 5 on a Likert 5-point scale. We translated the scale. The internal consistency reliability test revealed a Cronbach’s alpha coefficient of 0.880. The construct validity test showed that the Kaiser-Meyer-Olkin value was 0.873, and Bartlett’s test of sphericity produced an *x* value of 8908.7 (*P* < .01).

### 2.3. Statistical analysis tools

SPSS25.0 was used to perform statistical correlation analysis (work stress/psychological capital/social support/anxiety) and regression analysis (Layer1: anxiety/gender/grade, Layer2: anxiety/work stress/psychological capital/social support) on the data. The mediation path coefficients of the structural equation were analyzed using AMOS25.0 (Pathway 1: work stress—psychological capital—anxiety; Pathway 2: work stress—social support—anxiety).

## 3. Results

### 3.1. Test for common methods bias

This study adopted an online answer method with a largely homogeneous response environment, potentially resulting in artificial covariation between the predictor and effector variables, and affecting the scientific validity of the results. Therefore, in this study, Harman single-factor detection was conducted on the study data, with 13 factors having eigenvalues greater than 1. The first factor explained 20.13%, which was less than 40.00%; thus, there was no common method bias.

### 3.2. Descriptive statistics and linear correlation analysis of variables

Correlation analysis of clinical work stress, psychological capital, social support, and anxiety revealed a significant positive correlation between clinical work stress and anxiety (*r* = 0.47, *P* < .01), a significant negative correlation between clinical work stress and psychological capital and social support (*r* = −0.46, *r* = −0.21, *P* < .01), a significant negative correlation between psychological capital and anxiety (*r* = −0.56, *P* < .01), a significant positive correlation between psychological capital and social support (*r* = 0.12, *P* < .01), and a negative correlation between anxiety and social support (*r* = −0.43, *P* < .01), as shown in Table 2.

**Table 2:** Means, standard deviations, and correlations of variables.

Variable	$\bar{X}$ ± S	Clinical work stress	Psychological capital	Social support	Anxiety
Clinical work pressure	41.70 ± 3.58	1.00			
Psychological capital	29.25 ± 11.39	−0.46**	1.00		
Social support	18.75 ± .9.30	−0.21**	0.12**	1.00	
Anxiety	56.47 ± 6.52	0.47**	−0.56**	−0.43**	1.00

*P < .05,

** *P* < .01,

***P < .001, the same below.

### 3.3. Hierarchical multiple regression analysis of variables on anxiety

The predicted values were stratified by gender and grade as the first layer; clinical work stress, psychological capital, and social support as the second layer; and anxiety as the outcome variable. Stratified regression analysis was conducted using the SPSS process plug-in program. The results showed that gender negatively predicted anxiety (*t* = −3.51, *P* < .001), grade was not a significant predictor of anxiety (*t* = 1.63, *P* > .001), psychological capital and social support negatively predicted anxiety (*t* = −2.56, *t* = −8.41, *P* < .001), and clinical work stress positively and significantly predicted anxiety (*t* = 15.71, *P* < .001), as shown in Table 3.

**Table 3:** Hierarchical multiple regression analysis of variables (n = 321).

Layering	Outcome values	Predicted values	*b*	*t*	*R* ^2^	VIF	*F*
Layer 1	Anxiety	Gender	−0.11	−3.51***	0.06	1.00	27.98***
		Grade	0.03	1.63	0.08	1.00	
Layer 2	Anxiety	Clinical work stress	0.31	15.71***	0.41	1.20	137.12***
		Psychological capital	−0.25	−2.56***	0.38	1.17	
		Social support	−0.19	−8.41***	0.61	1.59	

### 3.4. Test of the mediating effect of psychological capital and social support

**Figure 2.**
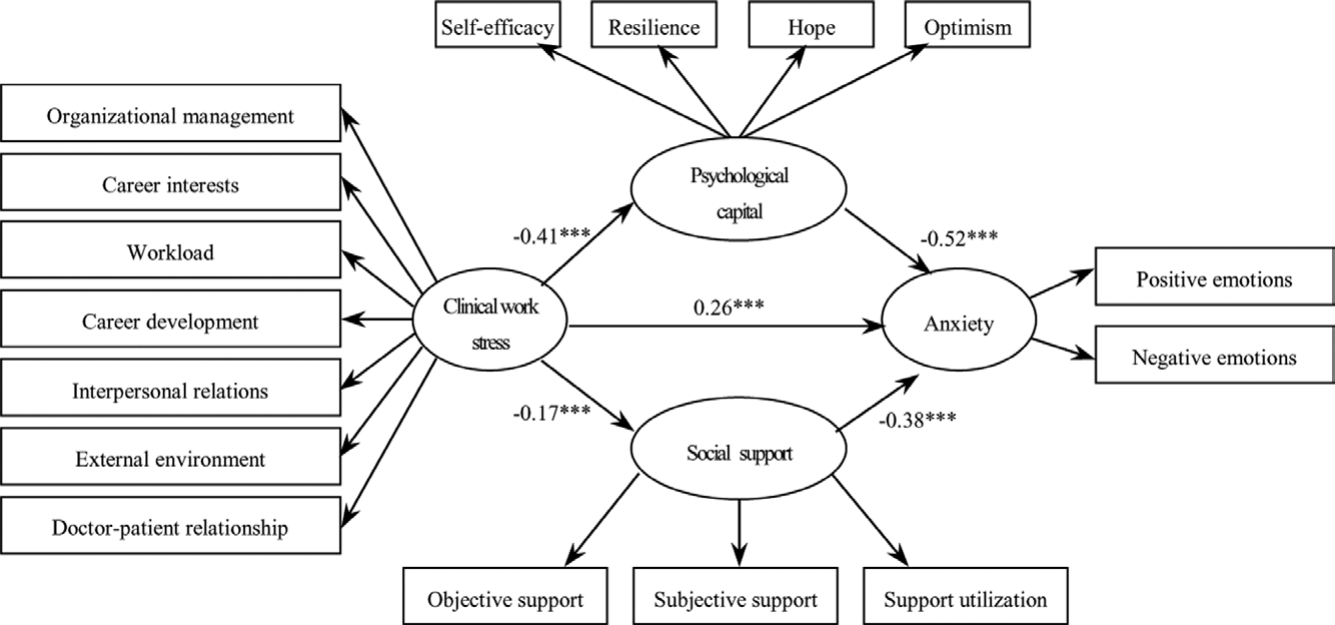
Multi-dimensional mediation model.

To further investigate the relationship between clinical work stress and anxiety among master’s degree nursing students, a structural equation model was used with clinical work stress as the independent variable, anxiety as the dependent variable, and psychological capital and social support as the mediating variables, as shown in Figure 2. To compare the agreement between the new model and the actual situation, the structural model was tested for the degree of fitness using AMOS structural equation analysis software. The absolute fit index of the new model c^2^/*df* = 1.48 (Fit Criterion < 3), Normed Fit Index = 0.94 (Fit Criterion > 0.9), and Parsimony Goodness-of-Fit Index = 0.67 (Fit Criterion > 0.5) were within the acceptable limits and the fit results were good.

The Bootstrap method was used to replicate the sample with a sample size of 5000; the results are listed in Table 4. Work stress effectively predicted psychological capital (*b* = −0.25, standard error [SE] = 0.04, *P* < .01, CI = [0.14, 0.42]), social support (*b* = −0.17, SE = 0.02, *P* < .01, CI = [0.08, 0.29]), and anxiety (*b *= 0.54, SE = 0.06, *P* < .01, CI = [0.45, 0.71]). Psychological capital effectively predicted anxiety (*b* = −0.52, SE = 0.06, *P* < .01, CI = [0.41, 0.65]) social support effectively predicted anxiety (*b* = −0.38, SE = 0.05, *P* < .01, CI = [0.29, 0.42]), and the 95% CI of the regression coefficient between the variables did not include 0. The indirect effect of work stress on predicting anxiety through psychological capital was statistically significant (effect value = 0.21, Boot SE = 0.04, CI = [0.19, 0.39]), and work stress was statistically significant in predicting anxiety through social support (effect value = 0.07, Boot SE = 0.01, CI = [0.02, 0.15]), with 95.00% CI for each path value excluding 0. Thus, psychological capital and social support had significant mediating effects on work-related stress and anxiety.

**Table 4:** Results of multiple mediating effect test.

Paths	Effect value	Boot SE	95% CI	
			Lower limit	Upper limit
Total effect	0.54		0.32	0.49
Direct effect	0.26	0.05	0.05	0.28
Total indirect effect	0.28	0.06	0.20	0.37
Pathway 1: Clinical work stress—psychological capital—anxiety	0.21	0.04	0.19	0.39
Pathway 2: Clinical work stress—social support—anxiety	0.07	0.01	0.02	0.15

CI = confidence interval, SE = standard error.

## 4. Discussion

This study showed that clinical work stress could directly affect anxiety, and high levels of clinical work stress lead to high levels of anxiety. In addition, clinical work stress could indirectly affect anxiety through psychological capital and social support, and psychological capital and social support could moderate anxiety.

### 4.1. The relationship between clinical work stress and anxiety

The results of this study suggest that work stress can positively predict anxiety; that is, the greater the work stress encountered by master’s degree nursing students in clinical work, the greater the possibility of generating anxiety, thus verifying Hypothesis H_1_. Studies have suggested that healthcare workers facing intense work stress during major epidemics may experience depression, anxiety, insomnia, and other stress-related symptoms.^[15]^ A study that focused on the physiological 5-HTTLPR gene polymorphism showed that individuals with the long allele were more likely to suffer from anxiety disorders than those with the short allele when exposed to work stress.^[16]^ Another study found that the prevalence of anxiety in the general population was 11.52%, while in graduate medical students, it was 27.54%, including 8.68% for severe anxiety.^[17]^ This study is not only consistent with existing research results but also focuses on the research perspective of a special group of master’s degree nursing students, who are engaged in heavy clinical front-line work of long duration, high intensity, and complex working environments. Due to the lack of proven anxiety assessments and intervention mechanisms, the anxiety of master’s nursing students has not been taken seriously. According to the conclusions of this study, medical schools and hospitals could take various steps to reduce the clinical work stress of master’s degree nursing students in order to lower the level of the anxiety which the students often feel.

### 4.2. The mediating role of psychological capital and social support

The results of the correlation analysis between psychological capital and anxiety showed that master’s degree nursing students with high levels of positive psychological capital had a low level of anxiety. The conclusion above is in line with the results which asserted that richer positive psychological capital of nursing postgraduates leads to a higher sense of meaning in life and a greater ability to cope with anxiety.^[18]^ Positive psychological capital could reduce symptoms of anxiety among nurses by alleviating job burnout.^[19]^ The results of this study also found that psychological capital has a significant positive effect on work stress. The study showed that individuals with abundant psychological capital are able to handle huge work stress with ease, and that rich psychological capital can compensate for the exhaustion caused by strong work stress. The conclusion above is in line with the results which asserted that psychological capital is an important resource for coping with work stress positively, compensating for the depletion of psychological resources and alleviating the stress response.^[20]^ Thus, psychological capital plays a mediating role in the relationship between clinical work stress and anxiety, verifying Hypothesis H_2_. In the clinical work of master’s degree nursing students, it is necessary to fully understand the mediating role of psychological capital in clinical work stress and anxiety. Nurses should enhance their positive psychological capital by self-efficacy, optimism, hope, resilience, forgiveness, and prosocial aspects to reduce anxiety.

The results of the correlation analysis between social support and anxiety indicated that richer social support of master’s degree nursing students resulted in lower anxiety levels. The conclusion above is in line with the results which assert that the higher the level of social support an individual receives, the lower the level of anxiety.^[21]^ Good social support helps people reduce social anxiety and lower the probability of depression.^[22]^ In addition, social support also has a significant positive impact on work stress; individuals who receive moral and material support from society and family were able to make use of social support to effectively alleviate the stress caused by work. This conclusion is in line with the results which asserted that social support can reduce individuals’ negative assessment of stress, enhance their intrinsic motivation to perform their duties, dilute the subjective experience of stress, and reduce the adverse effects of stress.^[23]^ Social support is an abundant resource that can moderate the effects of work stress.^[24]^ Thus, social support plays a mediating role in the relationship between clinical work stress and anxiety, confirming H_3_: Social support for master’s degree nursing students plays a mediating role between job stress and anxiety. Social support can be regarded as an important regulator of mental health in nursing students with a master’s degree. In the context of stressful clinical practice, methods that establish and improve the social support systems of master’s degree nursing students are important to effectively reduce anxiety.

### 4.3. The regression analysis of variables on anxiety

To further explore the relationship between clinical work stress and anxiety, psychological capital and anxiety, and social support and anxiety, a stepwise regression analysis was carried out with anxiety as the dependent variable. The 2 dimensions of demographic variables, gender and grade, were set as the independent variables to explore the predictive power of demographic variables on anxiety. As shown in Table 3, the dimensions of gender and grade were entered into the regression equation, which was significant. The results of the collinearity diagnosis indicated no collinearity problems. Therefore, the gender dimension had a negative predictive effect on anxiety ((*t *= -3.51, *P* < .001), and the grade dimension did not have a negative predictive effect on anxiety (*t *= 1.63, *P* > .001). A stepwise regression analysis was conducted with anxiety as the dependent variable and psychological capital and social support as independent variables to explore the predictive power of these variables on anxiety. As shown in Table 3, psychological capital and social support entered the regression equation, and the regression equation was significant. The results of the collinearity diagnosis indicated no collinearity problems. Therefore, psychological capital and social support had negative predictive effects on anxiety (*t *= −2.56, *t *= −8.41, *P* < .001). This may be because China is vigorously promoting medical system reform, and patients have increasingly strict requirements for medical quality. Medical personnel are under tremendous physiological and psychological pressure as a result, which makes them more prone to anxiety and depression.

### 4.4. Strengths and limitations of the research

This research has the following innovation: First, it used psychological capital and social support as mediating variables to analyze the relationship between clinical work stress and anxiety in master’s degree nursing students. To the best of our knowledge, this is the first such study. Second, this study used correlation and multi-layer regression analyses to explore the relationship between multiple variables and established a structural equation model to obtain data on the direct impact and mediation role. However, this study has some limitations, such as the small sample size. In future research, we will increase the sample size and improve the representativeness of the research sample to provide more references for the growth and development of master’s degree nursing students.

## 5. Conclusion

This study shows that clinical work stress can directly affect the anxiety of master’s degree nursing students but can be regulated through the mediating role of individual psychological capital and social support. Therefore, medical universities and internship hospitals should take practical measures, such as rationalizing internship rotations, improving the clinical environment, and upgrading the level of supervising teachers to reduce the triggers of anxiety among master’s students in nursing. They should also strengthen education for master’s degree nursing students so that they can demonstrate a positive psychological state during clinical practice, and provide them with more social support to counteract the anxiety triggered by clinical work stress.

## Author contributions

**Conceptualization:** Chunguang Ling.

**Data curation:** Chunguang Ling.

**Formal analysis:** Chunguang Ling, Shaojie Yu.

**Funding acquisition:** Chunguang Ling, Shaojie Yu.

**Investigation:** Shaojie Yu.

**Methodology:** Chunguang Ling, Shaojie Yu.

**Project administration:** Chunguang Ling.

**Resources:** Chunguang Ling.

**Software:** Shaojie Yu.

**Writing – original draft:** Chunguang Ling.

**Writing – review & editing:** Chunguang Ling.
